# Supplementary material for: Intuitive therapist robot patient physical interaction is worth a thousand words
Source: Sci Rep. 2026 Jul 21;16:33102. doi: 10.1038/s41598-026-63191-x (PMC13683037; doi:10.1038/s41598-026-63191-x)
Supplement: Supplementary file 1 [file 41598_2026_63191_MOESM1_ESM.pdf]

Supplementary-Materials: Intuitive Therapist Robot Patient Physical Interaction is Worth a Thousand Words

Beatrice Luciani, Alex van den Berg, Matti Lang, Alexandre L. Ratschat, and Laura Marchal-Crespo

1 Questionnaires

1.1 Participant Information Questionnaire

1. **Participant Number:** ____________________
2. **Name:** ____________________
3. **Age:** ____________________
4. **Sex assigned at birth:**
   - Male
   - Female
   - Other (specify): ____________________
5. **Is English your mother tongue?**
   - Yes
   - No (specify): ____________________
6. **What is your English level?**
   - A1 – Beginner
   - A2 – Elementary
   - B1 – Pre-intermediate
   - B2 – Intermediate
   - C1 – Upper-intermediate
   - C2 – Advanced
   - C3 – Native speaker
7. **Handedness:**
   - Right-handed
   - Left-handed
   - Ambidextrous
8. **Do you have previous experience with Immersive Virtual Reality?**
   - Never used
   - Used once or twice
   - Used occasionally (a few times a year)
   - Used frequently (several times a month)
   - Used very frequently
9. **Do you have experience with the Sigma.7 device?**
   - Never used
   - Used once or twice
   - Used occasionally (a few times a year)
   - Used frequently (several times a month)

- Used very frequently

10. **Do you have experience with the ARMin Exoskeleton?**
    - Never used
    - Used once or twice
    - Used occasionally (a few times a year)
    - Used frequently (several times a month)
    - Used very frequently
11. **Which role were you assigned for the experiment?**
    - Leader
    - Follower

1.2 Final IMI and NASA questionnaire

Intrinsic Motivation Inventory (IMI)

Role: Leader ☐ Follower ☐

Perceived Competence

Statement	Level of agreement						
	1	2	3	4	5	6	7
	Not at all true			Somewhat true			Very true
I think I am pretty good at this activity	○	○	○	○	○	○	○
I think I did pretty well at this activity, compared to other students	○	○	○	○	○	○	○
After working at this activity for a while, I felt pretty competent	○	○	○	○	○	○	○
I am satisfied with my performance at this task	○	○	○	○	○	○	○
I was pretty skilled at this activity	○	○	○	○	○	○	○
This was an activity that I couldn't do very well	○	○	○	○	○	○	○

Effort

Statement	Level of agreement						
	1	2	3	4	5	6	7
	Not at all true			Somewhat true			Very true
I put a lot of effort into this	○	○	○	○	○	○	○
I didn't try very hard to do well at this activity	○	○	○	○	○	○	○
I tried very hard on this activity	○	○	○	○	○	○	○
It was important to me to do well at this task	○	○	○	○	○	○	○
I didn't put much energy into this	○	○	○	○	○	○	○

NASA Task Load Index (NASA-TLX)

Instructions: Please indicate your rating for each dimension on a scale from 0 (Very Low) to 20 (Very High) by marking the bar.

Dimension / Question	Rating Bar (0–20)
Mental Demand: How mentally demanding was the task?	0 ____ 20
Physical Demand: How physically demanding was the task?	0 ____ 20
Temporal Demand: How hurried or rushed was the pace of the task?	0 ____ 20
Performance: How successful were you in accomplishing what you were asked to do?	0 ____ 20
Effort: How hard did you have to work to accomplish your level of performance?	0 ____ 20
Frustration: How insecure, discouraged, irritated, stressed, and annoyed were you?	0 ____ 20

2 Interview questions

Questions for the Trainer:

- Did you follow any strategy when using the Haptic device?
- Was the virtual environment useful or confusing?

Questions for the Trainee:

- Did you feel safe?
- Was the experiment physically challenging?

Questions for both the Trainer and the Trainees:

- Which condition did you prefer?
- Did you feel in control of the execution of the movements?
- Did you have any communication problems?
- Do you have any extra comments or remarks?

3 Statistical analysis results

Outliers were identified and removed using the following criterion: values below $Q1 - 2 \cdot \text{IQR}$ or above $Q3 + 2 \cdot \text{IQR}$, where $Q1$ and $Q3$ are the first and third quartiles, respectively. Across all dyads and conditions, 1.25-5.42% of observations were excluded per metric (see Tab. 1). Removal reduced the mean values by 6-14% and decreased the standard deviation, reflecting the effect of extreme values. The Wilcoxon rank-sum test indicated that the distribution of the remaining data was not significantly different from the original ($p > 0.05$), confirming that the central tendencies of the data were preserved.

Table 1. Outlier removal per metric and demonstration type. Percentages indicate the proportion of observations excluded using the 2.0· IQR criterion. Means and standard deviations are reported before and after outlier removal.

Metric	Condition	Original	Removed Out.	% Out.	Mean Before	Mean After	SD Before	SD After
Completion Time	VD	240	8	3.33	22.6	20.4	16.5	9.94
	HD	240	13	5.42	21.3	18.3	16.3	8.83
Smoothness	VD (Elbow)	240	7	2.92	-4.20	-3.92	2.44	1.83
	VD (Hand)	240	9	3.75	-3.95	-3.67	2.15	1.58
	HD (Elbow)	240	3	1.25	-3.87	-3.76	2.15	1.90
	HD (Hand)	240	4	1.67	-3.74	-3.62	2.02	1.82

Before selecting the final model for our analyses, we compared alternative formulations. In the final model, the effect of the different poses on the quantitative outcomes was not included, as it was not relevant for addressing the main research questions of the study. For completeness, however, we report here the models that were built including the factor **Pose**. When comparing these extended models with the final models presented in the paper, we found that removing **Pose** did not lead to a substantial increase in either the BIC or AIC indices.

3.1 Completion Time

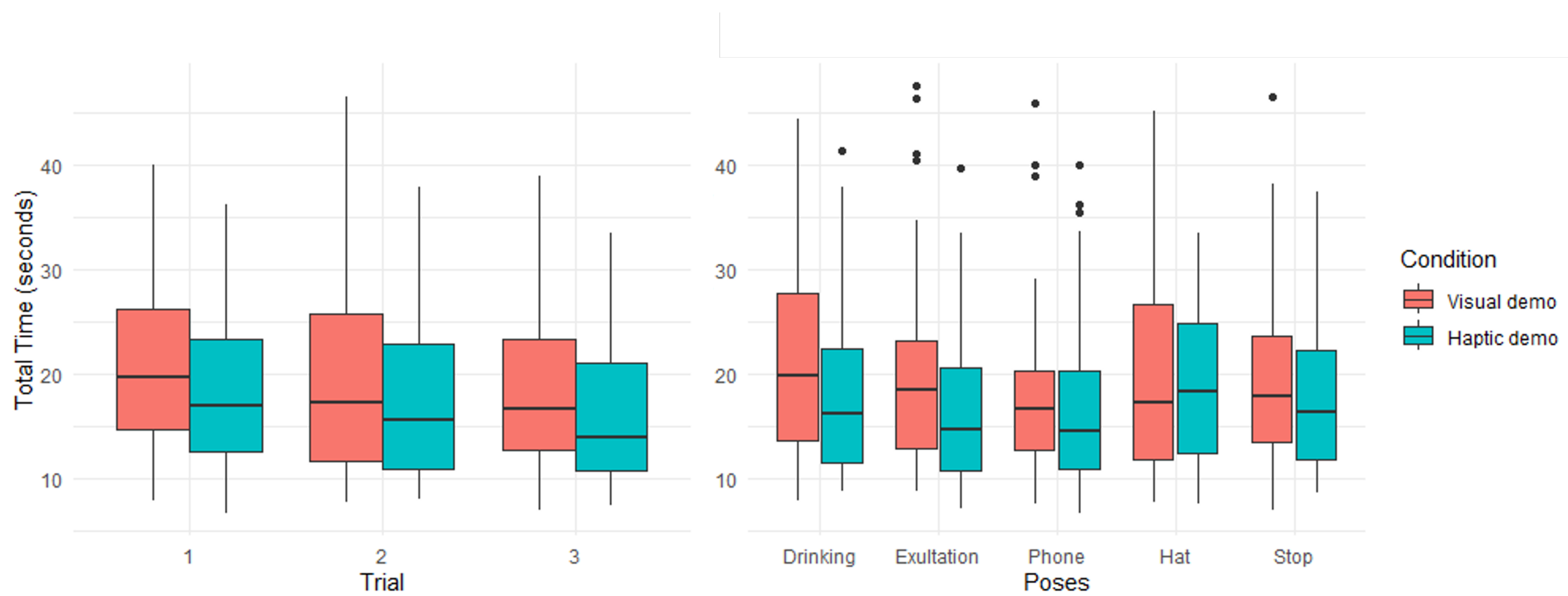

(a) Completion time –per trial.
(b) Completion time –per pose.

Figure 1. (a) Statistics of the time needed to complete the poses in the two conditions, along with the trials. (b) Statistics of the time needed to complete the poses in the two conditions, divided by pose.

	Estimate	Std. Error	df	t value	Pr(> |t|)	
(Intercept)	19.21	1.92	39.16	9.98	2.56e-12	***
Haptic	-3.43	1.89	421.23	-1.82	0.0698	.
Pose_Exult	2.15	1.53	421.13	1.40	0.1621	
Pose_Phone	3.33	1.54	421.17	2.16	0.0312	*
Pose_Stop	1.49	1.54	421.24	0.97	0.3350	
Pose_Hat	1.36	1.52	421.11	0.89	0.3716	
Order2	-0.16	2.21	17.22	-0.07	0.9430	
Trial	-0.89	0.60	421.28	-1.48	0.1399	
Haptic:Pose_Exult	-1.60	2.19	421.25	-0.73	0.4642	
Haptic:Pose_Phone	-1.19	2.20	421.20	-0.54	0.5879	
Haptic:Pose_Stop	0.03	2.19	421.10	0.01	0.9902	
Haptic:Pose_Hat	0.57	2.16	421.13	0.26	0.7933	
Haptic:Order2	4.58	1.40	421.20	3.28	0.00112	**
Haptic:Trial	-0.40	0.86	421.56	-0.46	0.6432	

Table 2. Results of the Completion Time LMM. *($p < 0.05$), **($p < 0.01$), ***($p < 0.001$)

3.2 Smoothness

	**Elbow smoothness [-]**						**Wrist smoothness [-]**					
	Estimate	Std. Error	df	t value	Pr(> |t|)		Estimate	Std. Error	df	t value	Pr(> |t|)	
(Intercept)	-4.18	0.68	32.96	-6.17	5.83e-07	***	-3.98	0.35	51.98	-11.54	5.95e-16	***
Haptic	1.23	0.64	461.39	1.92	0.056	.	0.13	0.35	460.31	0.36	0.723	
Pose_Exult	0.13	0.31	461.23	0.43	0.668		0.01	0.29	460.38	0.038	0.970	
Pose_Phone	0.06	0.31	461.23	0.20	0.839		0.48	0.29	460.39	1.652	0.099	.
Pose_Hat	0.20	0.31	461.23	0.65	0.515		0.34	0.28	460.26	1.188	0.235	
Pose_Stop	0.02	0.31	461.05	0.054	0.957		-0.046	0.285	460.17	-0.160	0.873	
Order2	-0.10	0.37	20.28	-0.278	0.784		0.030	0.368	19.43	0.082	0.935	
Trial	0.201	0.121	461.20	1.659	0.098	.	0.199	0.111	460.26	1.793	0.074	.
Haptic:Pose_Exult	0.095	0.448	461.42	0.211	0.833		0.566	0.405	460.28	1.399	0.163	
Haptic:Pose_Phone	0.009	0.443	461.17	0.019	0.985		0.230	0.404	460.19	0.568	0.570	
Haptic:Pose_Hat	-0.004	0.443	461.16	-0.008	0.993		0.505	0.400	460.19	1.262	0.208	
Haptic:Pose_Stop	0.119	0.442	461.09	0.269	0.788		0.337	0.399	460.13	0.843	0.400	
Haptic:Order2	-0.640	0.281	461.24	-2.280	0.023	*	-0.711	0.254	460.22	-2.801	0.005	**
Haptic:Trial	-0.005	0.173	461.20	-0.027	0.979		-0.015	0.156	460.18	-0.098	0.922	

Table 3. Results of the LMMs for the smoothness at elbow and wrist levels. *($p < 0.05$), **($p < 0.01$), ***($p < 0.001$), .($p < 0.1$)

4 Audio recordings analysis results

The speech classification pipeline was trained and evaluated on manually annotated data. The training set consisted of 46 labeled sentences (FEEDBACK: 9, INSTRUCTION: 20, NULL: 17), while validation was performed on an independent dataset of 162 sentences (FEEDBACK: 41, INSTRUCTION: 44, NULL: 77). On the testing set, the classifier achieved an overall accuracy of 75%. The corresponding confusion matrix (rows represent true labels and columns represent predicted labels) is reported below:

	INSTRUCTION	FEEDBACK	NULL
INSTRUCTION	44	0	12
FEEDBACK	6	38	13
NULL	1	12	52

Table 4. Confusion matrix reporting results of the testing of our sentence classifier. Rows represent true labels and columns represent predicted labels.

For the INSTRUCTION class, which is central to our analysis, precision was 0.86, recall was 0.79, and F1-score was 0.82.

5 Questionnaires results

According to the questionnaire results for the NASA-TLX, Trainers experienced a significantly higher mental demand than Trainees. The HD condition, however, did not significantly alter the mental demand of the global population compared to the VD condition.

In contrast, if being a Trainer did not significantly increase the perceived physical demand with respect to being a Trainee, Trainees globally perceived less physical demand during the HD condition. Lastly, our participants reported significantly lower perceived effort in the HD condition, with a non-significant effect for the role × condition interaction. Analyzing the temporal demand, performance, and frustration subcategories, we did not detect any significant effect due to the Role or the Condition.

Outcome	Term	Estimate	Std. Error	95% CI	df	Pr(> |t|)	p_{Holm}
**Mental Demand**	(Intercept)	22.50	5.27	[12.09, 32.91]	51.36	8.39e-05 ***	3.36e-04 ***
	Trainer	30.00	7.45	[15.82, 44.18]	51.36	1.86e-04 ***	3.73e-04 ***
	HD	-0.94	5.72	[-12.14, 10.26]	30.00	0.871	0.871
	Trainer:HD	-3.44	8.09	[-19.45, 12.57]	30.00	0.674	0.871
**Physical Demand**	(Intercept)	55.31	6.04	[43.47, 67.15]	59.25	6.00e-13 ***	2.40e-12***
	Trainer	-7.81	8.54	[-24.65, 9.03]	59.25	0.364	0.364
	HD	-20.31	8.05	[-36.05, -4.57]	30.00	0.017 *	0.034*
	Trainer:HD	14.69	11.38	[-7.70, 37.08]	30.00	0.207	0.275
**Temporal Demand**	(Intercept)	20.63	5.05	[10.76, 30.50]	43.19	1.86e-04***	7.45e-04***
	Trainer	10.94	7.14	[-3.08, 24.96]	43.19	0.133	0.265
	HD	4.06	4.38	[-4.53, 12.65]	30.00	0.361	0.481
	Trainer:HD	-2.50	6.19	[-14.67, 9.67]	30.00	0.689	0.689
**Performance**	(Intercept)	76.25	4.20	[68.01, 84.49]	55.98	<2e-16***	<2e-16***
	Trainer	-1.56	5.94	[-13.28, 10.16]	55.98	0.793	0.793
	HD	-2.81	5.08	[-12.83, 7.21]	30.00	0.584	0.779
	Trainer:HD	5.94	7.19	[-8.38, 20.26]	30.00	0.415	0.779
**Effort**	(Intercept)	53.13	5.78	[41.80, 64.46]	58.76	5.86e-13***	2.34e-12***
	Trainer	7.50	8.18	[-8.62, 23.62]	58.76	0.363	0.363
	HD	-28.44	7.56	[-43.28, -13.60]	30.00	7.36e-04***	0.001**
	Trainer:HD	18.75	10.70	[-2.35, 39.85]	30.00	0.090.	0.120
**Frustration**	(Intercept)	10.31	5.17	[0.11, 20.51]	60.00	0.0506.	0.201
	Trainer	12.19	7.31	[-2.33, 26.71]	60.00	0.101	0.201
	HD	1.88	7.31	[-12.52, 16.28]	60.00	0.798	0.798
	Trainer:HD	-4.06	10.34	[-24.33, 16.21]	60.00	0.696	0.798

Significance codes: *** $p < .001$, ** $p < .01$, * $p < .05$, . $p < .1$

Table 5. Linear Mixed Model results for NASA-TLX subscales.

Outcome	Term	Estimate	Std. Error	95% CI	df	Pr(> |t|)	p_{Holm}
**Perceived Competence**	(Intercept)	70.83	4.01	[62.99, 78.67]	38.67	$< 2 \times 10^{-16}$ ***	$< 2 \times 10^{-16}$ ***
	Trainer	-4.34	5.67	[-15.45, 6.77]	38.67	0.449	0.598
	HD	-0.69	2.42	[-5.44, 4.06]	352.00	0.774	0.774
	Trainer:HD	10.59	3.42	[3.89, 17.29]	352.00	0.002 **	0.004 **
**Effort**	(Intercept)	73.33	4.64	[64.22, 82.44]	42.79	$< 2 \times 10^{-16}$ ***	$< 2 \times 10^{-16}$ ***
	Trainer	5.00	6.56	[-8.87, 18.87]	42.79	0.450	0.450
	HD	-15.42	3.43	[-21.97, -8.87]	288.00	9.98e-06 ***	1.99e-05***
	Trainer:HD	11.88	4.85	[2.37, 21.39]	288.00	0.015 *	0.020 *

Significance codes: *** $p < .001$, ** $p < .01$, * $p < .05$, . $p < .1$

Table 6. Linear Mixed Model results for IMI questionnaire subscales.
